# Mesoporous Silica SBA-15 and SBA-15-NH_2_ as Potential Materials in the Adsorption of Glyphosate in Water Sources

**DOI:** 10.3390/nano16181186

**Published:** 2026-09-20

**Authors:** David Bocanegra-Cardenas, Carlos Alberto Guerrero-Fajardo

**Affiliations:** 1 Departamento de Química, Facultad de Ciencias, Universidad Nacional de Colombia, Cra 30 No. 45-03, Bogotá 111321, Colombia; dabocanegraca@unal.edu.co; 2 Departamento de Ingeniería Química, Facultad de Ingeniería, Universidad Nacional de Colombia, Cra 30 No. 45-03, Bogotá 111321, Colombia

**Keywords:** sustainability, SBA-15, adsorption, glyphosate, sustainable goals

## Abstract

Glyphosate is one of the most widely used herbicides in the agricultural sector. Its use worldwide is a cause for concern due to its harmful impact on water sources. Therefore, various techniques have been developed to remove this contaminant from water and rainwater. This research aims to produce SBA-15, whose name originated from the University of California, Santa Barbara (SBA), while the number 15 refers to the sample number, resulting in the material (SBA-15), which is a mesoporous silica made of silicon dioxide, an activated mesoporous silica, useful in adsorption processes thanks to its high porosity. Its mesoporous network is suitable for enzyme immobilization, making it one of the most widely used materials for contaminant adsorption. In this research, approximately 10.2 mg/g of glyphosate was removed at concentrations between 0 and 250 mg/L, and about 7.8 mg/g was removed using the activated carbon sample; this allows us to infer that two different samples are being compared. The contaminant was adsorbed with SBA-15, with the maximum adsorption rate achieved in water at a concentration of 250 mg/L. However, when SBA-15 is combined with NH_2_, the removal rate is even higher, reaching approximately 15.0 mg/g of contaminant removal. In both cases, a favorable fit greater than 0.99 is obtained using the Freundlich model. This research is significant due to the large number of water sources contaminated with this hazardous herbicide and, therefore, contributes to the UN Sustainable Development Goals.

## 1. Introduction

Glyphosate has a long history in the world because it was created with the aim of innovating agricultural processes due to a mid-20th century increase in agricultural production in the West. It was first produced in 1950 by a scientist who worked for the pharmaceutical company Cilag, although at that time the project was abandoned because it was not found to be useful in the pharmaceutical industry. Later, glyphosate was taken up as a cornerstone by Aldrich Chemical [1]. However, glyphosate was not patented as an herbicide until 1971, when it was introduced to the market in 1974 under the name Roundup. Despite its controversies, glyphosate has been one of the most widely used products in agro-commercial applications, as it has increased agricultural production around the world [2].

**Figure 1:**
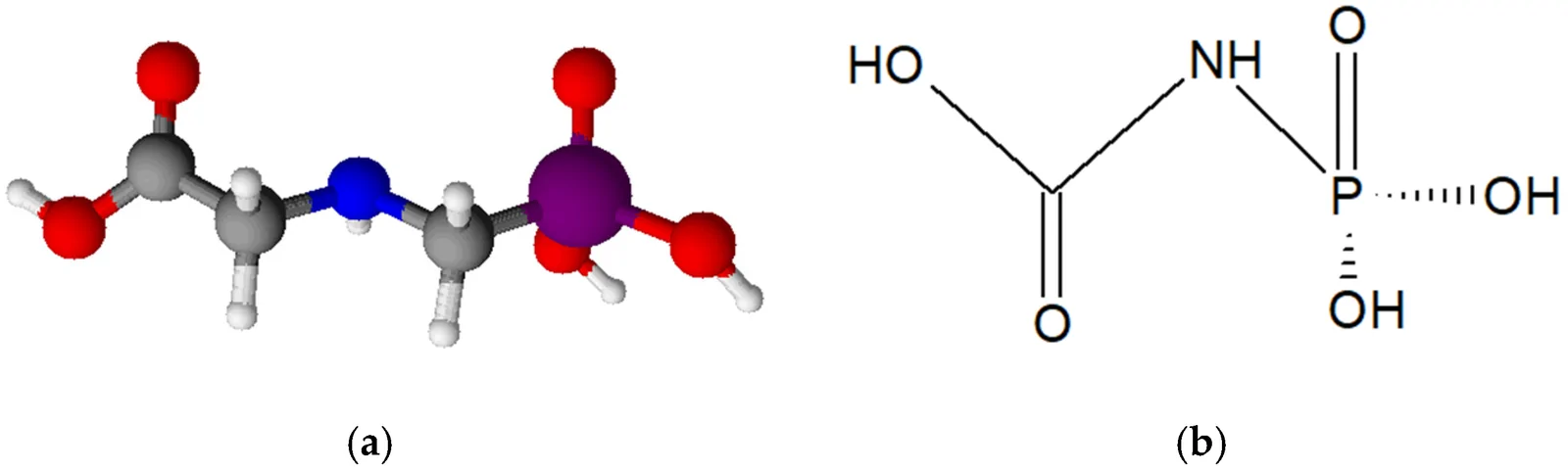
Chemical structure of glyphosate in (**a**). It is presented in the form of balls and bars in (**b**). It is presented in its semi-developed form.

Glyphosate (Figure 1) has widely been surrounded by controversies regarding its effects on human health and the environment; however, the field of research has diversified into multiple areas. Some countries with extensive herbicide production, including glyphosate, are the main producers of these substances: the USA, Brazil, Canada, China, and Argentina. Some of these are developed countries where the use of glyphosate has dramatically increased [2,3]. This increase is due to its high effectiveness and low cost in industrial agriculture; it is especially useful in the planting of genetically modified crops (corn and soybeans) [4].

Despite the above, glyphosate has been widely discussed, being the central topic of numerous investigations regarding health effects. An example of this is the daily exposure that people are subjected to, as glyphosate is a matter of debate due to its detection in the urine of workers and also in bodily fluids of individuals such as blood and breast milk, as well as in 60–80% of the population, including children. For the above reasons, in 2015 it was classified as a probable human carcinogen by the IARC (International Agency for Research on Cancer) [5,6].

Currently, multiple options exist for the adsorption and removal of herbicides (including glyphosate) from water sources, some of which are under investigation. For example, graphene is an innovative material for environmental cleanup, as it acts as a highly effective adsorbent and can be used in conjunction with filtration membranes. It is very useful for the adsorption of heavy metals, pharmaceutical chemicals, phenols, and other substances [7,8]. Other technologies have been in the commercial sector for some time. What is certain is that the current market is focused on producing selective herbicides; however, many of these adsorbent materials are too expensive. Therefore, it is important to replace them with new, innovative materials. Furthermore, SBA-15 offers a large surface area, large and uniform pores, and high structural stability, which facilitate the efficient capture of contaminants and large molecules.

This research will focus on an important material that has led to a growing interest in expanding the field of materials science. This material is SBA-15, which is a sustainable material that belongs to mesoporous silicas, an inorganic nanostructure characterized by a uniform arrangement of mesopores (pore size of 2–50 nm) within an amorphous silica matrix that presents a structure similar to that of a honeycomb [9]. The material SBA-15 has a wide range of applications due to its inherent properties, such as a large surface area, adjustable pore size, and promising physicochemical stability, which are useful in biological and engineering applications, such as drug delivery, biomedical devices, and sensors. Drugs have a high binding capacity with SBA-15, making it a good option for the development of biomedical devices [10].

It is important to mention that the future of SBA-15 is promising, as this material has the capabilities to take biomedical applications to the next level, specifically focusing on wound healing, bone regeneration, drug delivery, and skin tissue regeneration. Research has shown that an alteration of SBA-15 increases the drug adsorption capacity, as well as the thermal and mechanical stability of the material. Additionally, this material is promising for addressing global warming challenges with CO_2_ capture, which is one of the main problems in Environmental Engineering. It is basically common for SBA-15 to be synthesized using Pluronic 123 with a tetramethyl orthosilicate structure [10,11].

**Figure 2:**
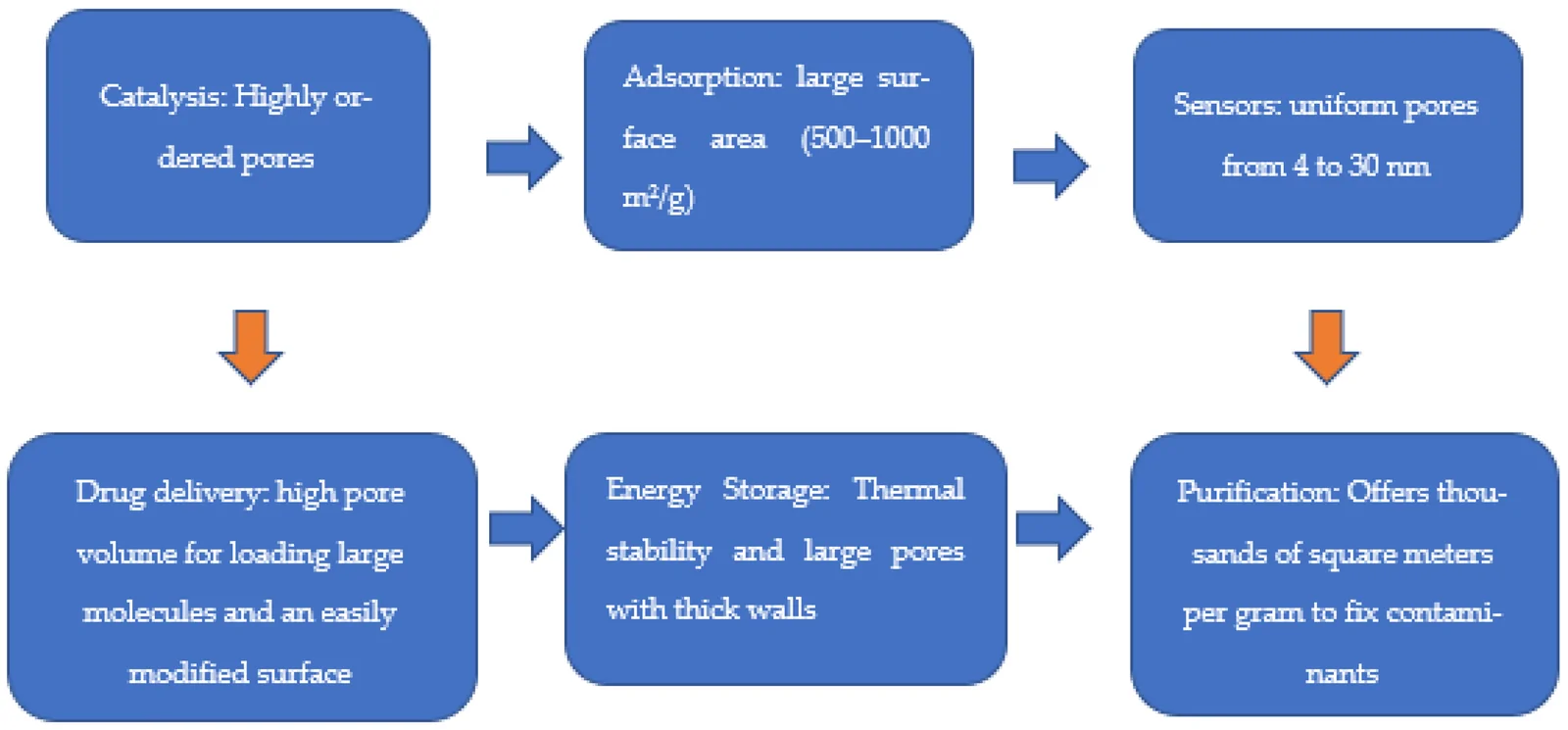
SBA-15 uses and applications.

**Figure 3:**
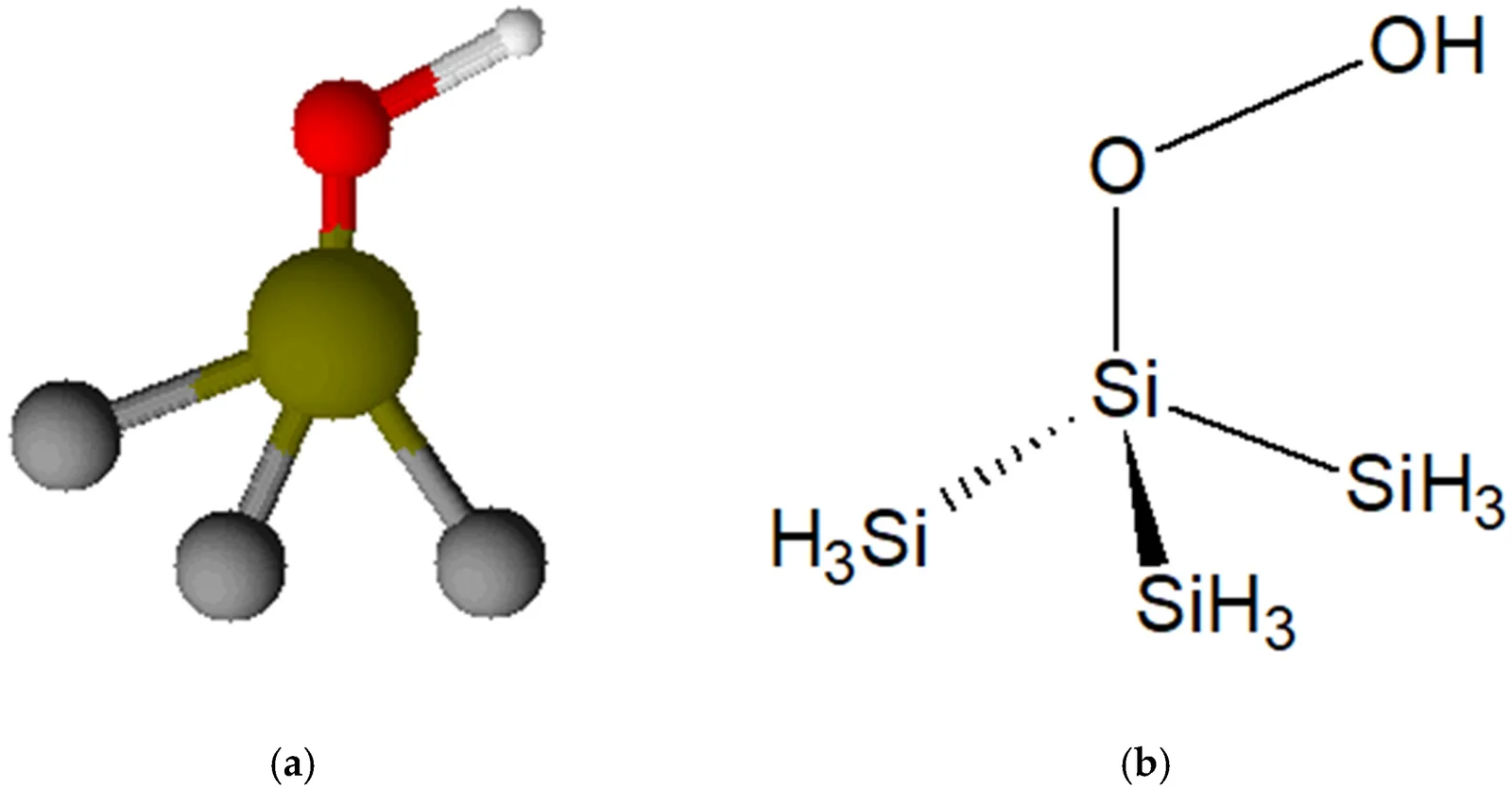
Silanol functional group in figure (**a**). Silanol group is presented in the form of balls and bars in (**b**). It is presented in its semi-developed form. The importance of presenting the structure lies in showing the bonds to observe the polarity, active sites, molecules, etc.

SBA-15, whose functionalities are clearly shown in Figure 2, is a silica powder that can be coupled using the sol–gel process. This material bonds to other materials primarily through its silanol (Si-OH) groups, whose molecule is shown in Figure 3. These silanol groups are the surfactants and the pathways by which this material can adsorb molecules and anchor metals, thus enabling the adsorption process. This allows it to act as a versatile support for catalysts, polymers, drugs, and enzymes [12]. Therefore, both the porous material and the material to which it is coupled are functionalized. In this coupling stage, other materials such as glutaraldehyde or glyoxal can be used as crosslinking agents, whose main function is to improve the mechanical and thermal resistance of the material through coupling [13].

The purposes of this research include investigating the maximum uptake capacities of the two materials and deriving indirect and direct information about the adsorption mechanism from adsorption isotherms modeling and FTIR measurements, respectively. This is because these two concepts are often confused and, despite sounding similar, are different. An adsorption process is the process of collecting materials in solution at a suitable interface where contaminants are adsorbed onto the surface of the adsorbent pores [14]. Adsorption penetrates the structure of the solid material, and the components penetrate the material’s structure. Absorption (with a “b”) refers to when one substance enters another, filling its entire volume. In this research, we are not discussing absorption because we want the contaminant molecules, specifically SBA-15, to be suspended on the wall. The absorption process (with a “b”) is similar to water absorbing water through a sponge. Adsorption (with a “d”), occurs when particles are trapped only on the surface of a material, like dust on a wall. We want to achieve this so that the solid material can be reused.

One way to mitigate the impact of the pollutant is by replacing mass spraying with controlled ground application or precision-focused drones to reduce chemical drift. This would allow for the use of new technologies, which contribute to strengthening voluntary substitution programs for illicit economies and reducing dependence on fumigation. Fumigation is also one of the main problems currently affecting vegetation and plants, creating a problem in both developed and developing countries.

**Figure 4:**
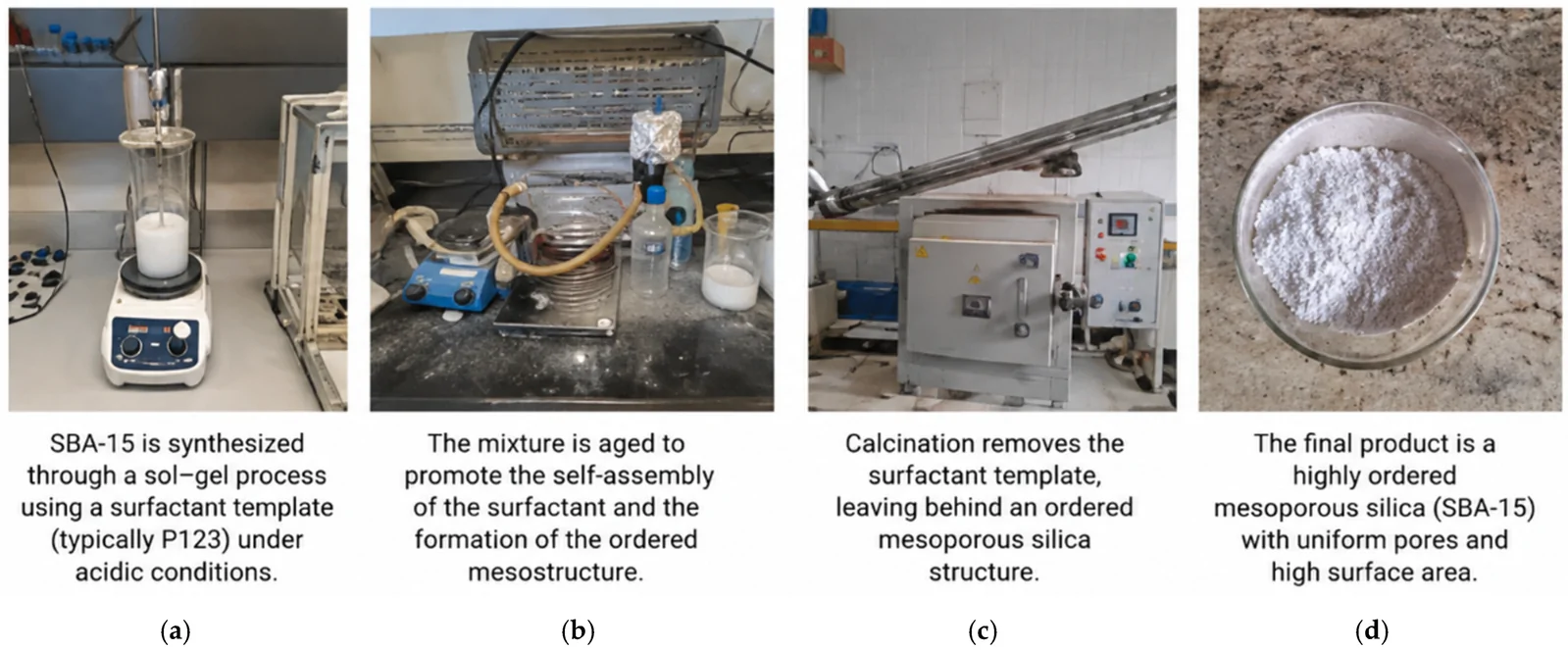
SBA-15 features and methodology. (**a**) shows the first step in the synthesis of SBA-15. (**b**) shows the filtration and mixing of SBA-15, which is carried out in a process known as hydro distillation. (**c**) shows the calcination in a muffle furnace. (**d**) shows the product obtained, which is a fine white powder.

The following is photographic evidence of the synthesis of SBA-15 and its different phases (Figure 4); it is important to present the photographic evidence of the experimentation since through this the reader could become familiar with the material and the process through which the adsorbent agent is obtained:

## 2. Materials and Methods

Table 1 below presents the method for preparing the material of interest SBA-15 and SBA-15-NH_2_:

### Physicochemical Characterization of SBA-15

**Raman Spectroscopy Characterization:** This research requires identifying the chemical composition and molecular structure through light scattering, since the aim is to analyze the surface of the porous material. On this occasion, the Thermo Fisher DXR 2 equipment (Cincinnati, OH, USA) is used, where a morphological analysis of the sample is performed.

**N2 adsorption and desorption isotherms:** This characterization is carried out in order to analyze the porous texture and specific surface area of solid materials (SBA-15 material). This physicochemical characterization technique is important because it allows for the standardization and characterization of surfaces. This is achieved by measuring the amount of gas that a material adsorbs and releases at a given temperature. In the present research, it is conducted using an Anton Parr physisorption equipment for adsorption analysis.

**Pore Size Distribution:** It is used to calculate the pore size distribution from nitrogen adsorption. Unlike classical methods such as BJH (which are based on the macroscopic Kelvin equation), QSDFT uses statistical mechanics at the molecular level to model how fluids condense or fill within the pores [17].

**Characterization with Fourier Transform Infrared Spectroscopy (FTIR):** In the present research, this technique is of great importance because it is used to identify and verify the chemical structure of materials by showing how the material adsorbs infrared light. This characterization is carried out using Anton Parr FTIR equipment.

## 3. Results

**Figure 5:**
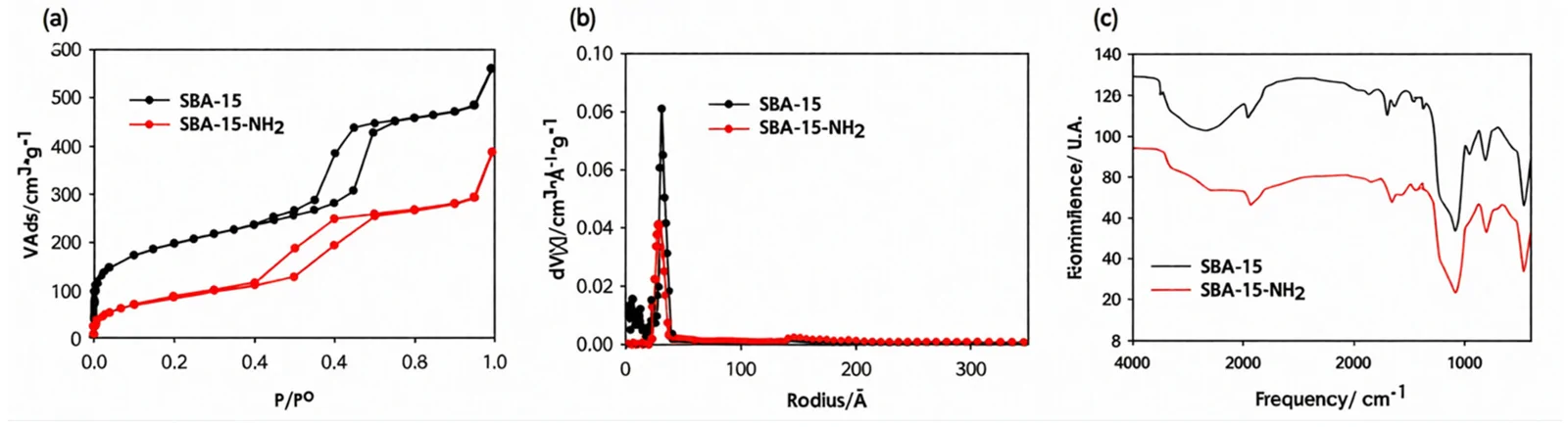
Textural parameters for the materials of interest for SBA-15 and SBA-15-NH_2_. In (**a**) the adsorption–desorption isotherms are presented, in (**b**) the peaks in functional bands and in (**c**) the bands and ranges for the functional groups.

Figure 5a shows the adsorption and desorption isotherms. More precisely, Type IV isotherms are characteristic of capillary condensation within mesopores, which means that these isotherms exhibit an initial monolayer–multilayer adsorption on the pore walls, followed by an abrupt change caused by capillary condensation. This indicates that increasing pressure leads to a change in the interaction of gas molecules with the solid surface, which is important in determining pore size.

The infrared spectra of the mesostructured silicas are shown in Figure 5c. Four characteristic bands were observed. The first band, located between 460 and 1090 cm^−1^, is typical of condensed silica networks and is associated with the Si-O-Si stretching vibration. The second band, located at 1600 cm^−1^ (exactly between 1630 and 1600 cm^−1^), indicates the presence of a primary amine and is associated with the bending vibrations of the -NH_2_ groups. The third band, located between 2870 and 2940 cm^−1^, is associated with the symmetric and asymmetric stretching vibrations of the C-H bond of the propyl chain of the anchoring group. These two bands demonstrate the APTES functionalization process. Finally, the band located at 3400 cm^−1^ is associated with the OH stretching vibration of the silanol groups, which favor the binding of glyphosate to the porous material. This band decreased in SBA-15-NH_2_, indicating that the 3-aminopropyltriethoxysilane molecule interacted effectively with the OH groups of SBA-15 [18,19]. The N_2_ adsorption–desorption isotherms were also obtained.

In Figure 5b, above this wavelength, a peak is obtained at 0.08 Å for SBA-15, which could indicate greater inhibition of the capture of polluting particles. This inhibition occurs in a band near 40 Å; from this, the chemical composition of the material can be determined.

Based on the results obtained in Figure 5 and Table 2, the textural parameters show that a larger pore diameter is obtained for SBA-15-NH_2_. However, it also indicates that SBA-15 alone allows for greater retention of dissolved contaminants in the material, thus improving the contaminant adsorption process. This is because the S_BET_ value is higher, meaning the material has a larger exposed surface area. Nevertheless, the average pore width is greater for SBA-15, which is an indicator of the adsorption process’s favorability, as pore sizes of 8.03 nm and 10.4 nm are obtained. However, this is not always indicative of a greater amount of contaminant adsorption. In this case, being very small (>2 nm), these pores offer the largest specific surface area [20]. Having a diameter very similar to that of the contaminant molecules, the attractive Van der Waals forces are multiplied, achieving the greatest adsorption capacity. Similarly, an average pore width of 31.6 Å and 29.4 Å is present. In this case, it is favorable for an adsorption process.

### Validation of Glyphosate Adsorption

**Figure 6:**
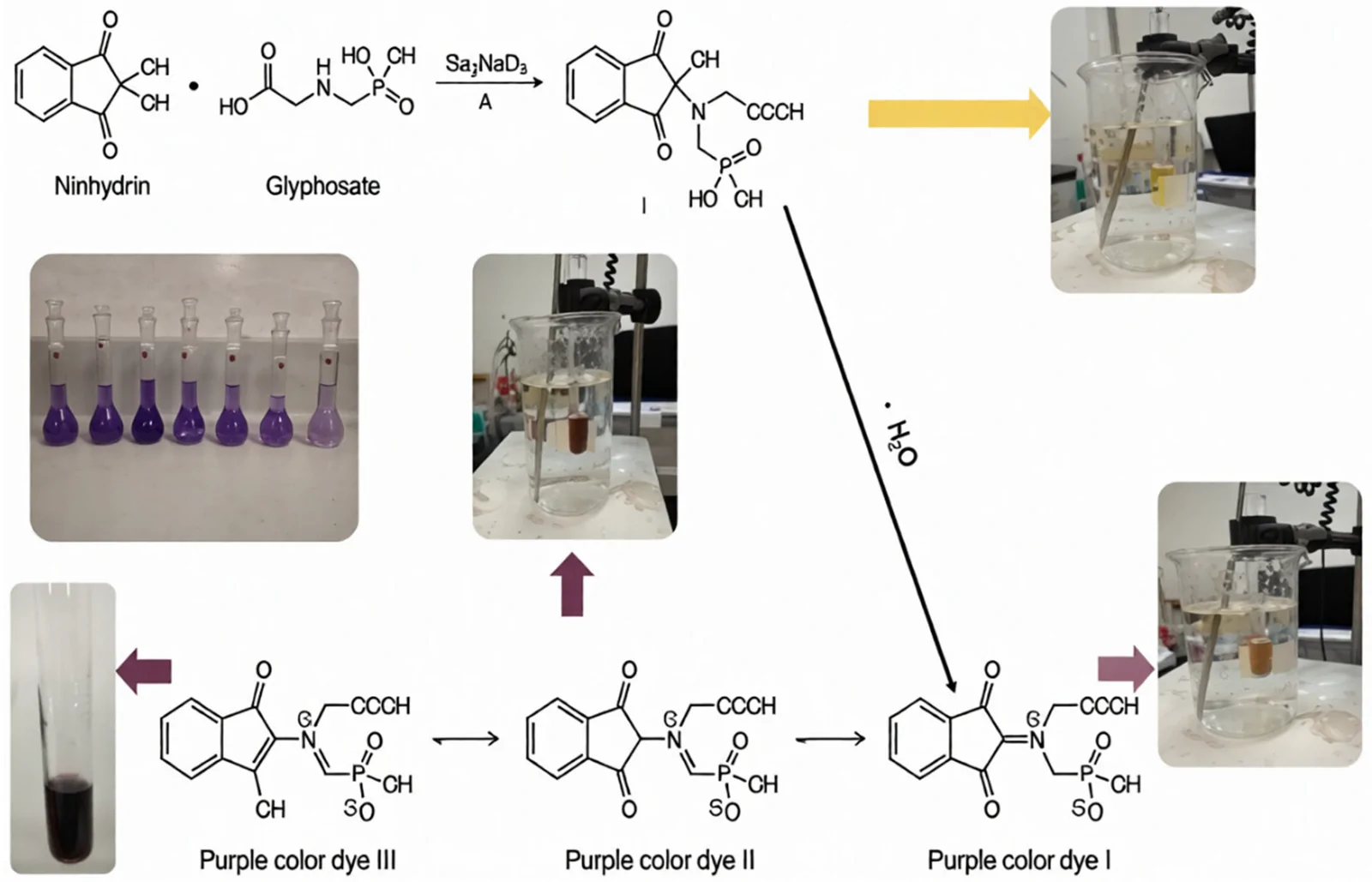
Photographic evidence of the glyphosate detection method in aqueous solution.

**Figure 7:**
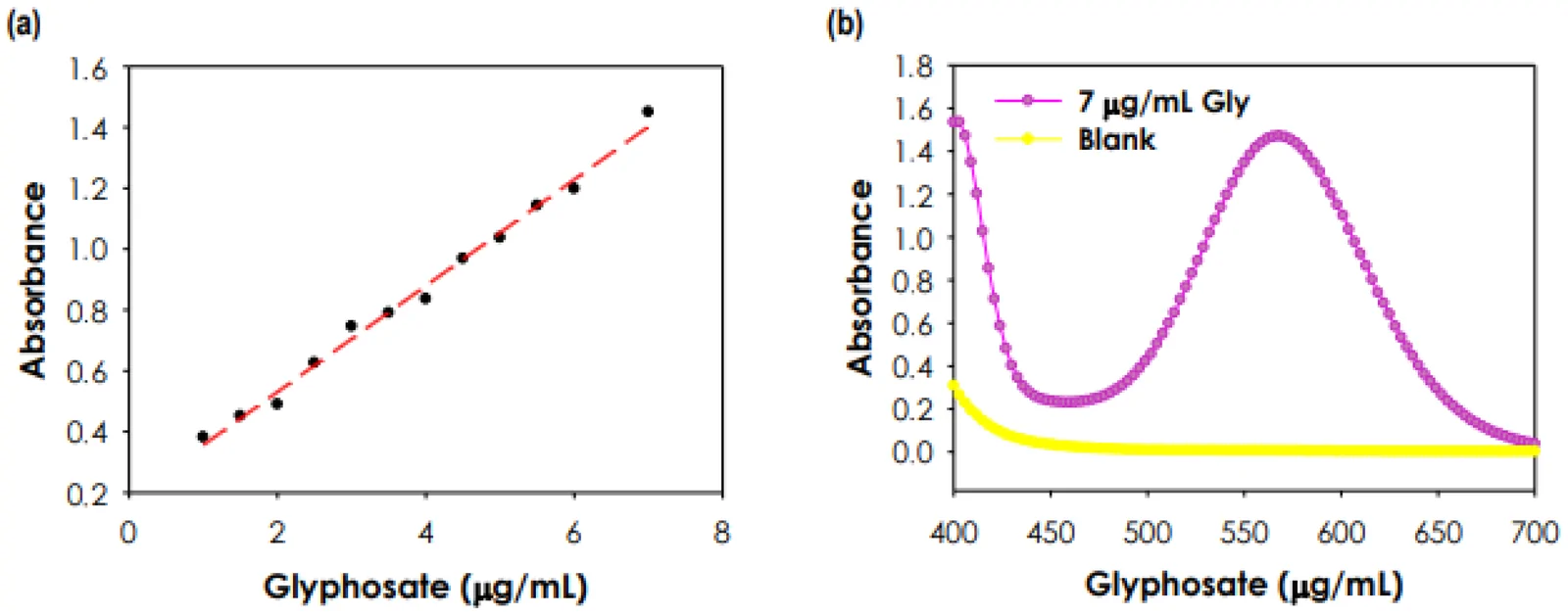
(**a**) Calibrati+on curve of glyphosate from aqueous solution by the ninhydrin method. (**b**) UV-Vis adsorption spectrum between 400 and 700 nm (original work).

The molecular size of adsorbates is a critical parameter for studying the adsorption capacity of solids with different textures, pore geometries, and pore distribution parameters. The molecular size of glyphosate has been estimated using molecular dynamics; the centroid distances that minimize interaction potentials are 6.395 Å, 7.410 Å, and 8.220 Å [21]. Therefore, based on the texture parameter evaluated for SBA-15 and SBA-15-NH_2_, restrictions on the diffusion of the glyphosate molecule in the mesoporous network are unlikely. However, restrictions could occur in the micropores formed in the walls (see Figure 6 and Figure 7). The obtained silica samples were tested as glyphosate adsorbents from aqueous solutions. Adsorption isotherms were measured by varying the initial concentration between 0 and 250 mg L^−1^.

**Figure 8:**
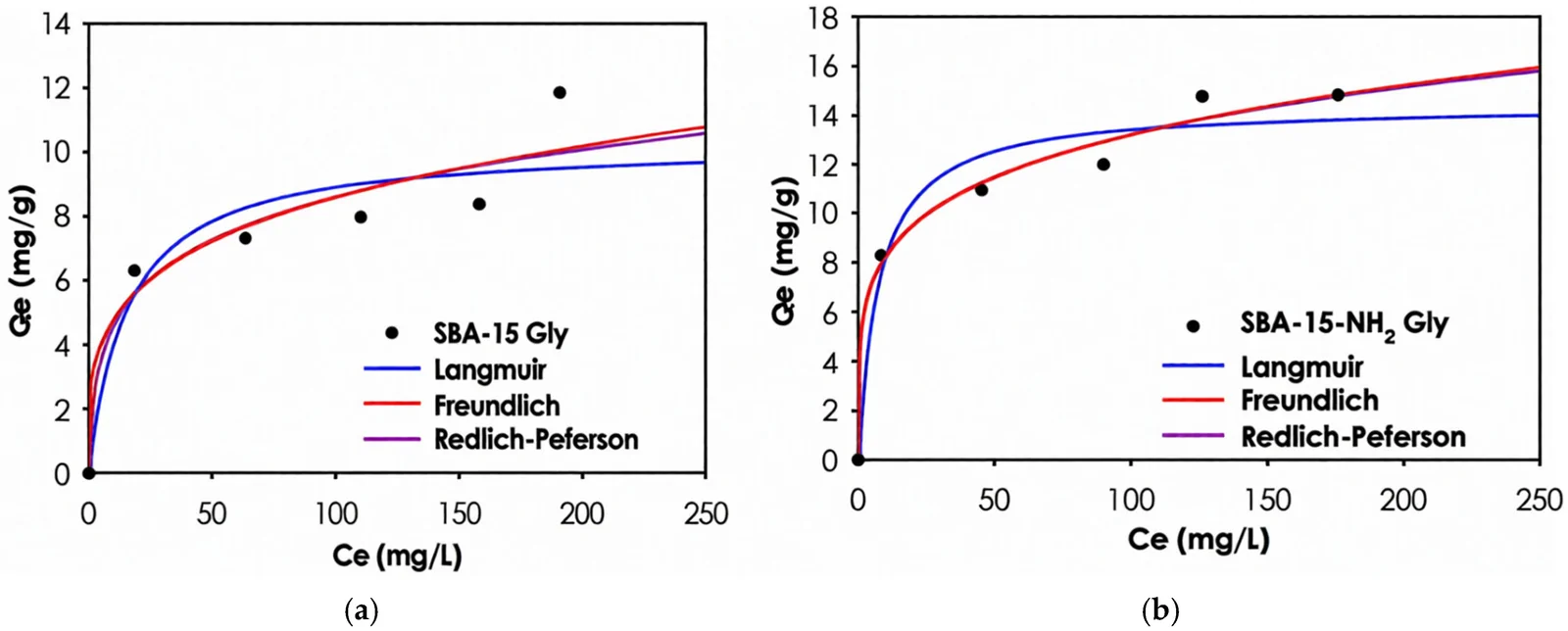
Glyphosate adsorption isotherms (**a**) SBA-15 (**b**) SBA-15-${NH}_{2}$.

The initial pH was not adjusted. The glyphosate isotherms for samples SBA-15 and SBA-15-NH_2_ are shown in Figure 8. Both Figure 8a,b show similarities in the adsorption processes, but SBA-15-NH_2_ is characterized by better adsorption, with an adsorption capacity of approximately 15.031 mg/g of contaminant, while SBA-15 only adsorbs 10.224 mg/g.

The results obtained from the characterization of SBA-15 revealed textural and chemical characteristics that suggest the suitability of this adsorbent for glyphosate removal. Furthermore, the adsorption interaction between glyphosate and SBA-15-NH_2_ is expected to occur through interaction with protonated amino groups via ion exchange and chelation mechanisms, as well as with the negatively charged functionalities of the herbicide [22,23]. Table 3 shows the best models that predict the adsorption of the contaminant. It can be seen that there is a better fit when using SBA-15-NH_2_ because its correlation coefficient is higher, approaching unity.

## 4. Discussion

The results obtained are even better than those of previous studies using activated carbon. As shown in Figure 8, an adsorption of approximately 10.2 mg/g is achieved, considerably lower than the 15.0 mg/g removed in this study. Even using only SBA-15, an adsorption of approximately 10.2 mg/g is achieved, similar to previously reported results. However, comparing this research with other studies is not considered relevant due to a lack of knowledge about the operating conditions and systems used in those studies. This study is limited to confirming that SBA-15 is, in fact, a potential agent for glyphosate adsorption and could be used in its removal process in water. This is extremely useful in the search for new materials, given the current need to solve problems such as water pollution. This can be achieved with new or little-explored materials such as SBA-15. Therefore, it is important not only for materials science but also for sustainability and the use and conservation of resources [24]. This is a call to the scientific community to improve sustainability processes in materials, a field still largely unexplored in the search for better ways to improve the quality of life for humans and animals.

**Figure 9:**
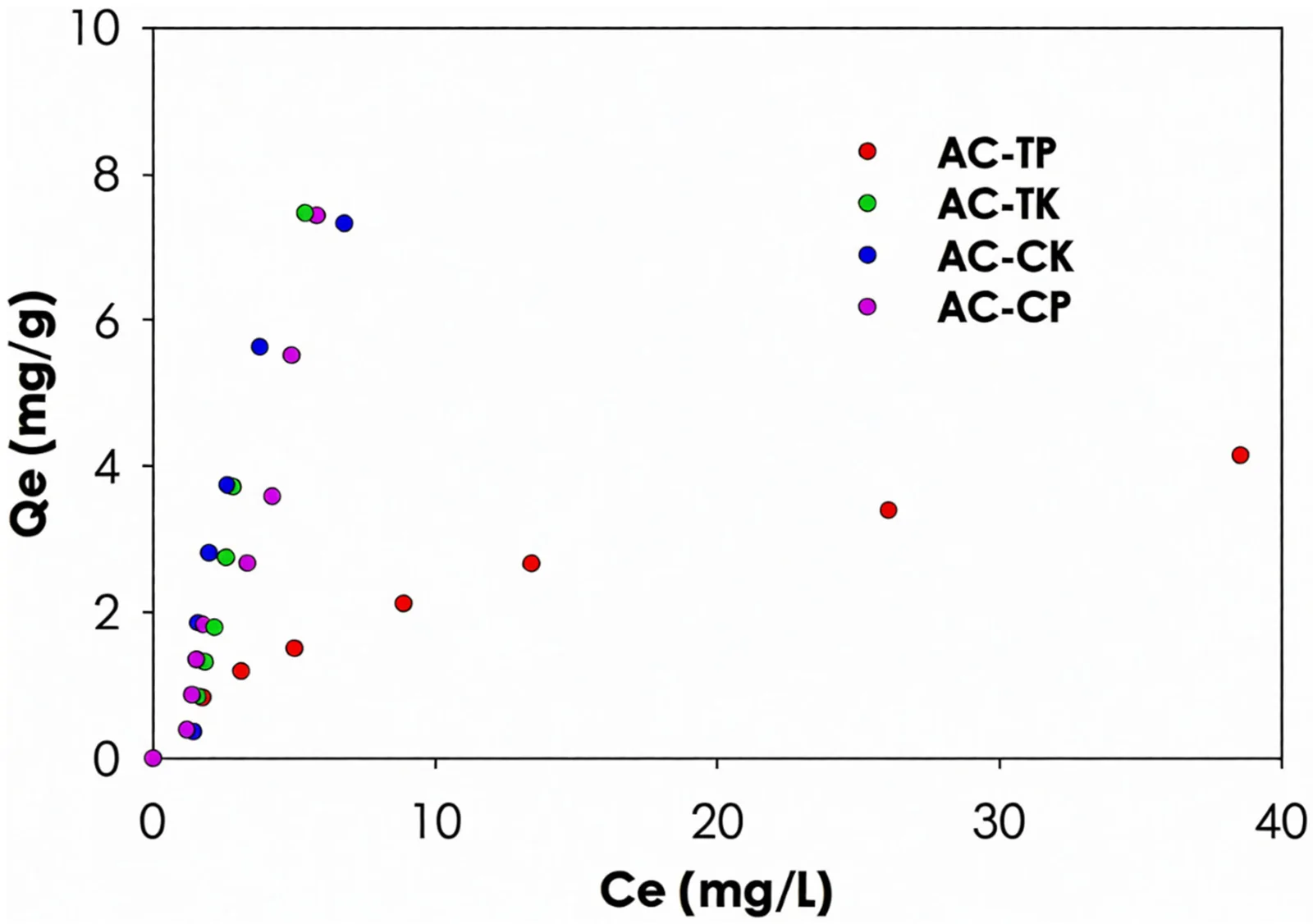
Glyphosate adsorption mechanism for each of the activated carbon samples [26].

If the present research is compared with previous research proposed by the authors, it is concluded that silica SBA-15 is more effective than activated carbon for high concentrations. According to Figure 9, the different samples and their adsorption capacity are shown, which, when compared to Figure 8, shows a lower adsorption capacity. However, this comparison should be taken with a grain of salt because the concentrations are not the same, but it also demonstrates its potential [25]. Four activated carbon samples are also shown to be effective materials in the adsorption of glyphosate.

In another investigation conducted on the capacity of iron to adsorb glyphosate, an adsorption of close to 34 mg/g of glyphosate was obtained. It is important to note that this was obtained according to the change in temperature, which is important because in the present investigation the change in temperature was not evaluated, which could change the adsorption capacity [26,27,28].

## 5. Conclusions

SBA-15 was prepared using the sol–gel method and modified by anchoring amine groups with APTES. The textural parameters were 593 and 302 m^2^/g, respectively. The decrease in these parameters is associated with surface functionalization. Regarding its chemical properties, spectrophotometric techniques revealed that the solids exhibit a heterogeneous surface chemistry, providing evidence of the APTES modification process. Glyphosate adsorption tests on SBA-15 showed Qo values of 10.2 and 15.0 mg/g for SBA-15 and SBA-15-NH_2_, respectively. This demonstrates the effect of surface chemistry and textural parameters on adsorption capacity. Additionally, the adsorption system on SBA-15 was studied by immersion calorimetry. The above is of great importance for the development of the Sustainable Development Goals, due to its significant impact on clean water and sanitation, as well as its role in fostering the development of functional materials capable of improving the quality of human life. This allows humanity to learn about new advances in materials.

SBA-15 is a mesoporous silica material that helps the environment by functioning as an advanced filter and support for cleaning water and air contaminants. It can adsorb toxic elements such as lead and arsenic in more than 95% of contaminated water, and it also serves as a support for degrading industrial dyes (such as methylene blue) and other organic waste.

## Figures and Tables

**Table 1:** Preparation method for SBA-15 and SBA-15-NH_2_

Mesostructured Silicas	Preparation Method
SBA-15	SBA-15 is synthesized by the sol–gel method, using Pluronic P123 as a structuring agent and tetraethyl orthosilicate (TEOS) as a silica source. The procedure is as follows: 18 g of Pluronic P123 are added to 135 g of water and stirred for 2 h. The mixture is then placed in a water bath at 35 °C, and a 2 M HCl solution and TEOS are added dropwise. The mixture is stirred for 20 h. Subsequently, the temperature is increased to 80 °C for 24 h. The precipitate is filtered from the mixture and washed with deionized water. Finally, the solid is calcined for 6 h at 540 °C [15,16].
SBA-15-NH_2_	2 g of SBA-15 were mixed with a solution of ethanol and (3-Aminopropyl) triethoxysilane (APTES), the mixture was brought to boiling temperature in a glass reflux system, after three hours the mixture was filtered and the solid was washed three times with ethanol, finally the solid remained in an oven at 80 °C overnight [15,16].

**Table 2:** Textural parameters of SBA-15 and SBA-15-NH_2_.

Samples	BET		DA (P/P° < 0.1)				QSDFT (P/P°$10^{-5}-1$)
	$S_{BET}$ $\left(m^{2}g^{-1}\right)$	C	$V_{mic}$ $\left(cm^{3}g^{-1}\right)$	$E_{O}$ $\left({KJmol}^{-1}\right)$	Pore Diameter (nm)	VP $\left(cm^{3}g^{-1}\right)$	Ancho Medio de Poro NLDFT (A)
SBA-15	593	100	0.25	12.5	8.03	0.87	31.6
SBA-15-NH_2_	302	250	0.09	16.2	10.4	0.58	29.4

**Table 3:** Model methods for SBA-15.

Samples	Two-Parameter Tuning Models						Three-Parameter Fitting Models			
	Langmuir Method			Freundlich Method			Redlich Peterson Method			
	$Q_{0}$	$K_{L}$	$R^{2}$	$K_{F}$	N	$R^{2}$	$K_{RP}$	$P_{E}$	b	$R^{2}$
SBA-15	10.256	0.0641	0.93923	2.6910	3.9833	0.96021	2.50895	0.72080	0.79565	0.95709
SBA-15-NH_2_	14.415	0.1322	0.97888	5.1258	4.8862	0.99369	388.84	73.080	0.80260	0.99384

## Data Availability

The original contributions presented in this study are included in the article. Further inquiries can be directed to the corresponding author.
